# Female homicides in the state of São Paulo, Brazil: a time series analysis from 1980 to 2022

**DOI:** 10.1590/1980-549720260031

**Published:** 2026-07-27

**Authors:** Edson Zangiacomi Martinez, Claudia Benedita dos Santos, Elisangela Aparecida da Silva Lizzi, Miriane Lucindo Zucoloto

**Affiliations:** I Universidade de São Paulo, Faculdade de Medicina de Ribeirão Preto – Ribeirão Preto (SP), Brazil.; II Universidade de São Paulo, Escola de Enfermagem de Ribeirão Preto – Ribeirão Preto (SP), Brazil.; III Universidade Tecnológica Federal do Paraná – Cornélio Procópio (PR), Brazil.

**Keywords:** Violence against women, Gender-based violence, Brazil, Homicide, Public health surveillance, Violência contra a mulher, Violência de gênero, Brasil, Homicídio, Vigilância em saúde pública

## Abstract

**Objective:**

This ecological study analyzes the temporal evolution of female homicide rates in the state of São Paulo, Brazil, from 1980 to 2022, using data from the Mortality Information System (SIM).

**Methods:**

Possible change points in the trend of these rates were identified using the Bayesian Estimator of Abrupt Change, Seasonality, and Trend (BEAST) model.

**Results:**

A total of 29,234 female homicide cases were recorded during the period, including 3,914 among young women aged 15 to 19. The analysis detected two major change points in 1995 and 2004, dividing the time series into three segments: an increasing trend until 1994, a stable period from 1995 to 2003, and a decreasing trend from 2004 onward. Among young women, three change points were identified, with a significant and consistent decline in homicide rates from 2004 to 2022.

**Conclusion:**

The number of female homicides in São Paulo has declined significantly since 2004, particularly among young women aged 15–19. Although this is linked to policy changes, the decline is likely due to multiple factors. The study highlights the importance of ongoing gender-focused policies and robust analytical tools in guiding public safety efforts.

## RESUMO

**Objetivo:**

Este estudo ecológico analisa a evolução temporal das taxas de homicídio feminino no estado de São Paulo, Brasil, de 1980 a 2022, utilizando dados do Sistema de Informações sobre Mortalidade (SIM).

**Métodos:**

Possíveis pontos de mudança na tendência dessas taxas foram identificados por meio do modelo Bayesian Estimator of Abrupt Change, Seasonality, and Trend (BEAST).

**Resultados:**

Um total de 29.234 casos de homicídio feminino foi registrado durante o período, incluindo 3.914 entre mulheres jovens de 15 a 19 anos. A análise detectou dois pontos de mudança importantes em 1995 e 2004, dividindo a série temporal em três segmentos: uma tendência crescente até 1994, um período estável de 1995 a 2003 e uma tendência decrescente de 2004 em diante. Entre as mulheres jovens, foram identificados três pontos de mudança, com um declínio significativo e consistente nas taxas de homicídio de 2004 a 2022.

**Conclusões:**

O número de homicídios femininos em São Paulo diminuiu significativamente desde 2004, principalmente entre as jovens de 15 a 19 anos. Embora isso esteja ligado a mudanças de políticas públicas, o declínio provavelmente decorre de vários fatores. O estudo destaca a importância de políticas contínuas com foco em gênero e ferramentas analíticas robustas para orientar os esforços de segurança pública.

## INTRODUCTION

According to a report by the World Health Organization (WHO), violence against women is not a new phenomenon, nor are the consequences for their physical, mental, and reproductive health^1^. However, the growing recognition that these acts violate women’s and girls’ rights, limiting their participation in society and damaging their health and well-being, is a more recent development^1^. This WHO report defines ‘violence against women’ as encompassing various forms of violence, such as intimate partner violence, rape, sexual assault, and other forms of sexual violence perpetrated by individuals other than partners. It also covers female genital mutilation, honor killings, and the trafficking of women^1^.

Brazil has implemented various initiatives to combat violence against women. In 2006, Law 11.340/2006 was introduced, also known as the Maria da Penha Law^2^, recognizing gender-based violence as a human rights violation rooted in inequality. Prior to this law, situations of violence against women were, in most cases, considered crimes of lesser offensive potential. The penalties for these crimes were often symbolic, such as the distribution of basic food baskets or community labor, which contributed to a feeling of impunity^3^. In 2015, the Brazilian government formally recognized ‘feminicide’ as a specific and aggravated form of homicide by including this term in Law 13.104/2015 in the penal code, thus categorizing it as a heinous crime^4^.

Despite legislative advances, Brazil remains one of the most violent countries for women^5^. A study using data from the 2019 National Health Survey showed that 19.38% of Brazilian women reported experiencing violence^6^. Psychological violence was the most common subtype, both in isolation and in conjunction with other subtypes. Furthermore, the Global Burden of Disease Study on interpersonal violence against women showed that Brazil’s homicide rates among women aged 15 to 49 remained consistently high between 1990 and 2019, indicating an inability of conservative Brazilian society to protect women^7^.

This article uses time series analysis to examine annual female homicide rates in São Paulo from 1980 to 2022, based on data from the Brazilian Mortality Information System (SIM, in Portuguese). For the purposes of this article, ‘female homicide’ refers to the murder of women in the broadest sense^8^. This includes cases of femicide, which are related to gender issues, as well as cases involving general delinquency. As the coding of mortality data in the SIM follows the International Classification of Diseases and is based on death certificates, which do not specify the circumstances of the crime, analyzing data on female homicides can provide insight into how these cases have evolved within the Brazilian population.

## METHODS

### Study design and data source

This ecological and descriptive study is based on data from the SIM. It considers the annual number of female homicides in São Paulo, Brazil, from 1980 to 2022, categorizing deaths according to place of residence. This period was chosen based on the availability of consolidated data and the fact that it is long enough to make inferences. São Paulo is Brazil’s most populous and developed state, accounting for around 22% of the country’s population. The study used place of residence as a basis for analysis, since it more accurately reflects the social and cultural environment in which women live. This includes structural inequities and the availability of local protection and support systems, all of which can affect their susceptibility to violence.

In view of the potential demographic changes resulting from a reduction in the proportion of young people in the population over the period, the analyses considered homicides in all age groups, as well as among those in the final phase of adolescence (15–19-year-olds). Although this age group does not account for the largest proportion of female homicide victims in Brazil^5^, it is characterized by a high incidence of reported interpersonal and self-directed violence. In 2002, the rate of interpersonal and self-directed violence was reported as 707.1 cases per 100,000 inhabitants for women aged 15–19, compared to 585.8 and 503.0 cases for the 20–24 and 25–29 age groups, respectively^9^.

Up to 1995, female homicides were identified using codes from the 9th Revision of the International Classification of Diseases (ICD-9): E960–E978 (homicides and intentional injuries to other people) and E990–E999 (legal interventions). After 1995, the following ICD-10 codes were considered: X85–Y09 (aggression) and Y35 (legal intervention). To calculate the mortality rates, population projections for each federative unit by sex for each year, as well as population counts, provided by the Brazilian Institute of Geography and Statistics (IBGE), were used as the denominator.

Brazil is the fifth largest country in the world by area, spanning a territory of 8,514,876 square kilometers. São Paulo, located in the Southeast region, is the most populous state in Brazil, accounting for around a fifth of the country’s population. As this study used secondary data and no individuals were identified, it was not submitted to a research ethics committee. This is in accordance with national and international legislation regulating research involving humans.

### Statistical analysis

The change point analysis (CPA) method includes statistical tools used to investigate potential changes occurring within a series of data points. The Bayesian Estimator of Abrupt Change, Seasonality, and Trend (BEAST) is a flexible CPA method that detects abrupt changes (i.e., change points), cyclic variations, and nonlinear trends in time series observations^10^. Consider a time series denoted by (t_i_, y_i_), where i = 1,...,n, and y_i_ are observations of a variable at the time t_i_. In a general manner, BEAST assumes that this time series comprises three components: trend (*T*), seasonality (*S*), and change point, in addition to a noise term. The global model is thus given by Equation 1:


(1) $$y(t_{i},\Theta )=T(t_{i},\Theta_{\tau })+S(t_{i},\Theta_{s})+e_{i},i=1,\ldots ,n$$

where:

Θ_T_: parameter that specify the trend factor;

Θ_S_: parameter that specify the seasonal factor;

These parameters implicitly encode the abrupt change points. By “abrupt”, the authors of the BEAST algorithm refer to “any turning points or breakpoints at which trend or seasonal signals start to deviate from the previous regular trajectories”^10^. The method thus calculates the probability of a change point (cpPr), occurring at each year of the study period, and the probability distribution of the number of change points in the trend component. The mode of this distribution corresponds to the optimal number of change points. Further details can be obtained from the original article by Zhao et al.^10^.

In the present study, the presence of seasonal factors was not considered. For estimating the unknown parameters, the Bayesian approach was adopted, where the Markov Chain Monte Carlo (MCMC) sampling technique was employed to obtain random samples for posterior inference. Three MCMC chains were simulated, each with 100,000 samples. The first 1,000 samples from each chain were discarded to avoid the effect of the initial values. This analysis was performed on R (version 4.4.1) using the “Rbeast” package.

In the time series intervals in which the BEAST model suggests an approximately linear trend, it is possible to calculate the annual percentage change (APC)^11^. This is calculated using the Equation 2:


(2) $$\mathrm{APC}=(e^{b}-1)100\%$$

Where:

e: base of the natural logarithm;

b: angular coefficient of the Prais-Winsten regression model.

This analysis used the “prais” package in R.

#### Data availability statement:

The entire data set that supports the results of this study has been published in the article itself and in the ‘Supplementary Materials’ section.

## RESULTS

From 1980 to 2022, a total of 29,234 cases of female homicide were reported in the state of São Paulo, of which 3,914 were among women aged 15 to 19. No female deaths due to legal interventions were recorded until 1995 (ICD-9 codes E990–E999). After that year, 22 deaths (ICD-10 code Y35) were identified. This indicates that the majority of deaths were caused by homicides and violence.

**Figure 1:**
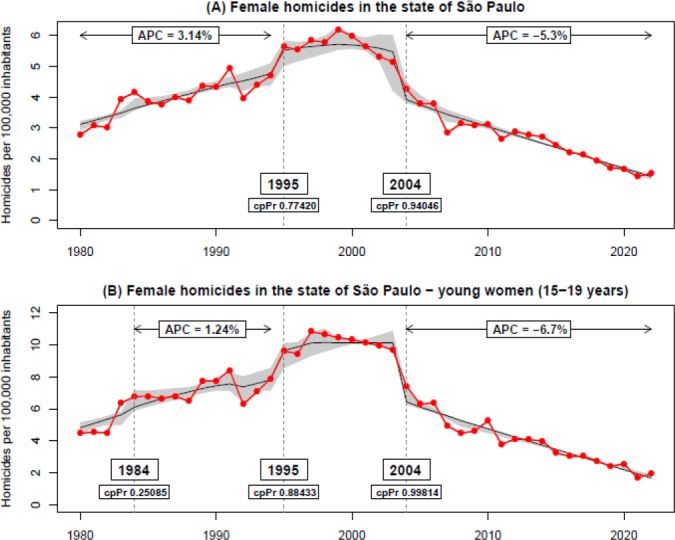
The red line shows the observed annual homicide rate for (A) all female age groups and (B) young women (aged 15–19) per 100,000 inhabitants in the Brazilian state of São Paulo, from 1980 to 2022. The black line shows the trend estimated using the BEAST algorithm, and the shaded area represents a 95% credible interval. The dashed vertical lines indicate the change points, and cpPr denotes the corresponding probability of occurrence. APC denotes the annual percentage change.

APC: annual percentage change; cpPr: probability of a change point;

Figure 1 (panel A) shows the results of using the BEAST algorithm to perform sequence decomposition and changepoint analysis on the time series of female homicide rates in the state of São Paulo, where a 69.1% probability was found that the trend component has two change points. The results indicate two abrupt change points occurring in 1995 and 2004, with respective cpPr of 0.9405 and 0.7742. These two change points divide the trend component within the time series into three segments. The first segment shows an approximately linear increase from 1980 to 1994, with rates ranging from 2.78 to 4.69 homicides per 100,000 inhabitants and an APC of 3.14% (95% confidence interval [CI] 1.72–4.59%). The second segment shows an increase from 1995 to 1999, when the homicide rate peaked at 6.18 per 100,000 inhabitants, followed by a decrease until 2003. In a visual interpretation of the graph in Figure 1, if a horizontal line segment is fully included within the 95% credible interval represented by the shaded area, as occurred between 1995 and 2003, then there is no statistically significant upward or downward trend during that period (similar to p<0.05). Finally, the third segment shows a linear decrease from 2004 until the end of the series. In 2004, the rate of female homicides was 4.25 per 100,000 inhabitants, and by 2022, this rate had fallen to 1.53, the lowest rate recorded during the entire study period (APC -5.3%; 95%CI -6.2 to -4.4%).

BEAST: Bayesian Estimator of Abrupt Change, Seasonality, and Trend.

Figure 1 (panel B) shows the results of applying the BEAST algorithm to the time series of homicide rates among young females (aged 15 to 19) in the state of São Paulo. It was found that there is a 53.3% probability of three change points in the trend component: in 1984 (cpPr 0.2509), 1995 (cpPr 0.8843) and 2004 (cpPr 0.9981). Between 1984 and 1994, the rate of homicides per 100,000 people increased from 6.76 to 7.89, with a non-significant APC of 1.24% (95%CI -0.59–3.11; p=0.17). Between 1995 and 2022, the rate varied relatively little. In 1995, the rate was 9.61 homicides per 100,000 people. An observable peak of 10.84 homicides per 100,000 inhabitants was reached in 1997, followed by a decrease until 2003, when the rate equaled 9.70 homicides per 100,000 inhabitants. However, no significant APC was observed between 1995 and 2022 (APC 0.07%; 95%CI -1.79–1.96%; p=0.95), indicating that the rates remained relatively stable during this period. From 2004 to 2022, there was a reduction from 7.42 to 1.93 homicides per 100,000 inhabitants, with an APC of -6.7% (95%CI -7.53 to -5.86%).

These results are summarized in Table 1. Details of the results of the BEAST analysis and the R codes are provided in the Supplementary Material.

**Table 1:** Annual percentage change (APC) for each time interval defined in the Bayesian Estimator of Abrupt Change, Seasonality, and Trend (BEAST) analysis.

Period	APC (%)	95%CI	p-value	Homicides per 100,000 inhabitants (beginning and end of the period)
**All female age groups**				
1980–1994	3.14	(1.72–4.59)	<0.01	2.78–4.69
1995–2003	-1.03	(-3.29–1.27)	0.33	5.63–5.14
2004–2022	-5.3	(-6.2 to -4.4)	<0.01	4.25–1.53
**Young women (aged 15–19)**				
1980–1983	1.38	(1.37–1.39)	<0.01	4.47–6.36
1984–1994	1.24	(-0.59–3.11)	0.17	6.76–7.89
1995–2003	0.07	(-1.79–1.96)	0.95	9.61–9.70
2004–2022	-6.7	(-7.53 to -5.86)	<0.01	7.42–1.93

## DISCUSSION

This study highlights a significant decrease in female homicide rates in São Paulo, particularly since 2004 and among young women. While other researchers have also observed this reduction^12,13,14,15,16,17.18^, the present article provides new insights. First, it demonstrates the practicality of the BEAST method for trend analysis using accessible software like R. Second, it updates earlier findings with an extended time series (1980–2022), in line with the current data availability in official systems. A third contribution is its focus on female homicides. Although the findings do not specifically address femicide, this focus enables a more targeted discussion grounded in gender-specific dynamics and legislation, while considering vulnerabilities often overlooked in analyses of the general population.

Analyzing public safety indicators in São Paulo municipality from 1996 to 2008, Peres et al.^12^ propose three main explanatory hypotheses for the homicide reduction: investment in public safety, socio-economic changes with improved quality of life, and demographic changes with a reduction in the proportion of young people in the population. Supporting the public safety investment hypothesis, Goertzel and Kahn^13^ suggest that the figures in São Paulo city and state between 2001 and 2007 may be attributable to more effective policing, such as the stricter enforcement of gun control.

The authors also noted that, in October 2003, the federal government introduced legislation restricting firearm imports, prohibiting unregistered gun ownership and public carrying, while also increasing penalties for violations of gun control laws. This legislation also established a weapons buy-back program involving federal and municipal governments and civil society^19^. Between July 2004 and October 2005, 470,000 guns were voluntarily surrendered in Brazil for up to $100 each. At the time, this was considered the second-largest buy-back campaign ever carried out worldwide^19^. Additionally, Peres et al.^14^ report a 169.5% increase in public security investment in São Paulo state between 1997 and 2008, with a 219.3% rise from 1997 to 2001 alone. Nadanovsky^15^, meanwhile, attributed the decrease in homicide rates in São Paulo state to an increase in incarceration, noting that the number of prisoners per 100,000 inhabitants had risen from 182 in 1996 to 341 in 2005.

In addition to the possible impact of changes in the demographic composition of the population on the reduction in homicide mortality rates in São Paulo state, the following factors, which are not related to public safety, have been identified: demographic changes, particularly a decrease in the proportion of young people; investment in social policies with subsequent socioeconomic improvements and enhanced quality of life; greater social participation through consolidated civil society initiatives; a declining unemployment rate; and state investment in education and culture^14,16,17,18^. Another possible explanation is heightened control by organized criminal groups over local crime^20,21,22^. The consolidation of a faction’s hegemony over the criminal underworld would have helped to establish peace in the markets for drugs, arms, vehicles, and contraband, as well as the legal markets associated with them^20^. This would have reduced conflicts and, consequently, the number of homicides.

All cited articles help to explain the general homicide decline since 2004. However, except for Martins et al.^18^, the ecological studies^12,13,14,15,16,17^ focused on the general population of São Paulo, rather than specifically on female homicides. In this specific context, some key events aligning with the beginning of the decline in homicide rates in 2004 include the launch of the Plano Nacional de Políticas para as Mulheres (National Plan for Policies for Women), stemming from the I Conferência Nacional de Políticas para as Mulheres (First National Conference on Policies for Women), which prioritized coordinating and expanding support networks^23^. Also in 2004, a consortium of feminist organizations and experts proposed domestic and family violence legislation, emphasizing the need for comprehensive laws and public policies. These efforts resulted in the Maria da Penha Law, sanctioned in 2006. This law marks a turning point in Brazil’s efforts to protect women, introducing tools to prevent abuse, ensuring stronger punishment for offenders, and offering support and protection to women in vulnerable situations^24^.

Nearly two decades after its enactment, the Maria da Penha Law remains central to protecting women in Brazil. This study’s analysis shows that its 2006 enactment did not cause an abrupt shift in São Paulo’s female homicide trend. However, significant national mobilization in support of the law had begun by 2004, and the subsequent updates, including provisions for immediate arrest, preventive detention, tougher sentences, and protective measures, have likely reinforced the steady decline in rates observed since that year.

In addition to these legal protections, in March 2015, the Feminicide Law No. 13,104/2015 was enacted, classifying feminicide as an aggravated form of homicide defined as the killing of a woman due to her gender. The law specifically recognizes cases involving domestic and family violence, or acts of contempt or discrimination based on the victim’s gender^24^. However, similar to the Maria da Penha Law, the Feminicide Law did not result in an immediate shift in São Paulo’s female homicide trend in the analysis herein. While legal protections may affect mortality trends and other outcomes, this change is not expected to be immediate. This is particularly true, given the limitations of the public system in enforcing the laws and ensuring citizen safety. Thus, using time series to assess the impact of laws on mortality rates can be challenging, although the enactment of the Feminicide Law is undeniably important in classifying cases of domestic and family violence or contempt and discrimination against the female condition as a criminal conduct, thereby sustaining the gradual decline in homicide rates within the state.

The identification of a change point in the time series in 1997 coincides with the period immediately following the implementation of ICD-10 in Brazil. In the case of SIM, the transition from ICD-9 to ICD-10 was not merely a change in codes, but an institutional effort to improve data quality, which reduced the use of ill-defined causes. Additionally, this change point coincides with the peak of the ‘homicide epidemic’ in the state of São Paulo in the late 1990s^25^. This period was characterized by intense urban violence and the consolidation of organized crime, which disproportionately affected young people. The high rates among women aged 15–19 in 1997 also highlight the absence of specific protective legislation at the time. The Maria da Penha Law, which aimed to address domestic and gender-based violence, and the Disarmament Statute, were only enacted in 2006 and 2003, respectively^2^.

Some authors have discussed the possibility that the COVID-19 pandemic may have contributed to an increase in violent crimes against women in various contexts and geographical regions^26,27^. According to Marques et al.^28^, factors linked to social isolation that contributed to the increase in these crimes include the victim’s isolation, making her more vulnerable, the aggressor’s use of alcohol or illicit drugs, which intensifies violence, greater ease for the aggressor to control the victim, and unemployment. Astudy by Nunes et al.^29^ showed that the mortality rate due to intentional violent crimes against women in Alagoas, Brazil, was higher in 2020 (5.33 per 100,000 people) than in the years prior to the pandemic. Other Brazilian studies, such as that by Leite et al.^30^, show that psychological, physical, and sexual violence perpetrated by intimate partners during the pandemic was widespread among women living in Vitória, in the Brazilian state of Espírito Santo. However, this trend does not appear in the data analyzed herein.

This study has several potential limitations. Firstly, and perhaps most importantly, the mortality rates found in this study may be biased as a result of underreporting issues, as well as the fact that they are based on population projections provided by the IBGE for each year, which may differ from actual census data. Another limitation lies in the fact that it was not possible to ascertain the type of homicide, the circumstances surrounding it, or the weapon used. For example, analyzing homicides by type of weapon can reveal the potential effect of firearm possession legislation on violent deaths. Cerqueira and Lins^31^ highlight the possibility of there being a number of “hidden homicides” within the mortality information system, i.e., homicides that are not officially recorded as such. To address this issue, the authors propose using machine learning techniques to estimate the probability that a violent death from an undetermined cause was actually a homicide. However, this strategy was not employed in the present study. Furthermore, when considering the number of unrecorded homicides in the country, it is necessary to consider cases where there is no death certificate, for example, when perpetrators conceal their victims’ bodies. This could also introduce bias into the results of this study, particularly given that the occurrence of this type of homicide may have increased over the period considered.

Despite these limitations, the study also presents some strengths. The temporal coverage of over four decades (1980–2022) enables the identification of long-term trends and structural shifts in female homicide rates, and it includes recent data not typically available in earlier studies, extending the analysis beyond previous research, which often ends in the early 2000s or 2010s^12,13,14,15,16,17,18^. Using the BEAST model is a methodological strength because, compared to more conventional linear models (e.g., joinpoint regression), BEAST can detect nonlinear trends and multiple change points. It also allows for a probabilistic interpretation of changes and accommodates model uncertainty in trend estimation. The results obtained can inform evidence-based public policy regarding violence against women.

In conclusion, this study describes a substantial and enduring decrease in the rate of female homicides in the Brazilian state of São Paulo, particularly since 2004, with a more pronounced reduction observed among adolescent females aged 15 to 19. Although the observed trends coincide with major legislative and policy developments, female homicide is a complex phenomenon influenced by multiple interrelated factors. These factors may include demographic changes, socio-economic improvements, shifts in criminal dynamics, civil society engagement, and evolving gender norms. While the statistical analysis provides evidence of significant temporal shifts, it does not allow for causal inferences. Nevertheless, the study highlights the importance of continued investment in gender-sensitive policies and emphasizes the value of advanced analytical tools for monitoring public health and safety indicators in complex social contexts.
