# Supplementary material for: Female homicides in the state of São Paulo, Brazil: a time series analysis from 1980 to 2022
Source: Rev Bras Epidemiol. 2026 Jul 27;29:e260031. doi: 10.1590/1980-549720260031 (PMC13440504; doi:10.1590/1980-549720260031)
Supplement: Supplementary file 1 [file 1980-5497-rbepid-29-e260031-supp1.pdf]

## Supplementary Material to: Female homicides in the state of São Paulo, Brazil: a time series analysis from 1980 to 2022

This document provides supplementary material to our paper "Female homicides in the state of São Paulo, Brazil: a time series analysis from 1980 to 2022" which was published in the Revista Brasileira de Epidemiologia (2026). It contains further details on the methods of analysis and results that are summarized in the main report.

### The BEAST method

The BEAST approach is a common method to decompose time series into trend and seasonality and for detecting abrupt changes in these two components. It has been used in a wide range of applications, including studies of land surface temperature[1], climate change detection[2] and the dynamics of infectious diseases[3]. Compared to joinpoint regression, which is currently widely used in time series studies in epidemiology, the BEAST approach may be more suitable for evaluating the dynamics of some health events, given that in the joinpoint approach, each segment between the change points is linear[4]. In contrast, the BEAST approach can detect nonlinear trends between successive change points using functions such as spline smoothing. In this study, BEAST was shown to be suitable for describing the behavior of female homicide data series, enabling significant periods to be identified.

### Statistical details

The Bayesian model averaging time-series decomposition algorithm (BEAST) is a parametric regression method developed by Zhao et al.[5]. It can capture various types of uncertainty, identify abrupt shifts of any size and reveal intricate nonlinear patterns in time series data. Let $\mathcal{D} = \{t_i, y_i\}_{i=1,\ldots,n}$ the observed data, where $y_i$ denotes observations of a variable at $n$ time points $t_i$. The statistical model for the time series $y(t_i)$ is given by

$$y(t_i) = S(t_i;\mathbf{\Theta}_S) + T(t_i;\mathbf{\Theta}_T) + e_i,\ i = 1,\ldots,n,$$

where the parameters $\mathbf{\Theta}_S$ and $\mathbf{\Theta}_T$ are estimated from the data $\mathcal{D}$, and they also implicitly encode the abrupt change points. We assume that the noise $e_i$ is Gaussian with a magnitude of $\sigma$, capturing the remainder of the data that is not explained by the seasonal

($S$) and trend ($T$) components[5,6]. To evaluate the unknown parameters, the algorithm employs Bayesian inference.

The R package `Rbeast` can be used to perform this analysis. Consider `wt` a data frame containing the variables `wt$ANO` (years from 1980 to 2022) and `wt$SP` (the female homicide rates in São Paulo from 1980 to 2022).

```
# Data
wt <- data.frame(
  ANO = 1980:2022,
  SP  = c(2.78, 3.07, 3.03, 3.94, 4.15, 3.85, 3.76, 4.00, 3.88,
          4.37, 4.34, 4.94, 3.97, 4.39, 4.69, 5.63, 5.54, 5.83,
          5.78, 6.18, 5.98, 5.64, 5.32, 5.14, 4.25, 3.78, 3.80,
          2.84, 3.14, 3.08, 3.13, 2.65, 2.89, 2.78, 2.72, 2.46,
          2.21, 2.14, 1.95, 1.72, 1.67, 1.43, 1.53))
```

The BEAST model can be fitted to data in the following way:

```
# Bayesian changepoint detection algorithm
library(Rbeast)
myts <- ts(wt$SP, start=c(1980), end=c(2022))
out <- beast(myts, season='none',
       dump.ci = TRUE, mcmc.burnin = 1000, mcmc.samples = 100000)
plot(out)
print(out)
```

In this R code, `library(Rbeast)` is used to load the `Rbeast` package, and the `ts` function is used to create the time-series object `myts` containing `wt$SP`. The `beast` function in the `Rbeast` package is then used to fit the BEAST model to the data, where the argument `season='none'` is used to specify that the seasonal component $S(t_i;\mathbf{\Theta}_S)$ will not be used in our analysis, and `dump.ci = TRUE` is used to create credible intervals for the estimated trend component. The argument `mcmc.burnin = 1000` specifies the number of burn-in samples discarded at the start of each chain (the default number of MCMC chains is three), while `mcmc.samples` specifies the number of samples to be collected per MCMC chain.

Figure S1 shows the results obtained from the `beast` function. The dashed vertical lines mark the most likely locations of changepoints and the green curve of Pr(tcp) shows the point-wise probability of changepoint occurrence over time. The "order$_T$" curve provides an estimate of the mean order of the piecewise polynomials required to adequately fit the trend (the 0-th order is constant and the 1st order is linear). An average order toward 0

means that the trend is more likely to be flat and an order close to 1 means that the trend is linear. "slpSign" are the probabilities of the trend slope being positive (red part), zero (green), or negative (blue). Finally, "error" are the residual errors between the observed and BEAST-modelled values.

.

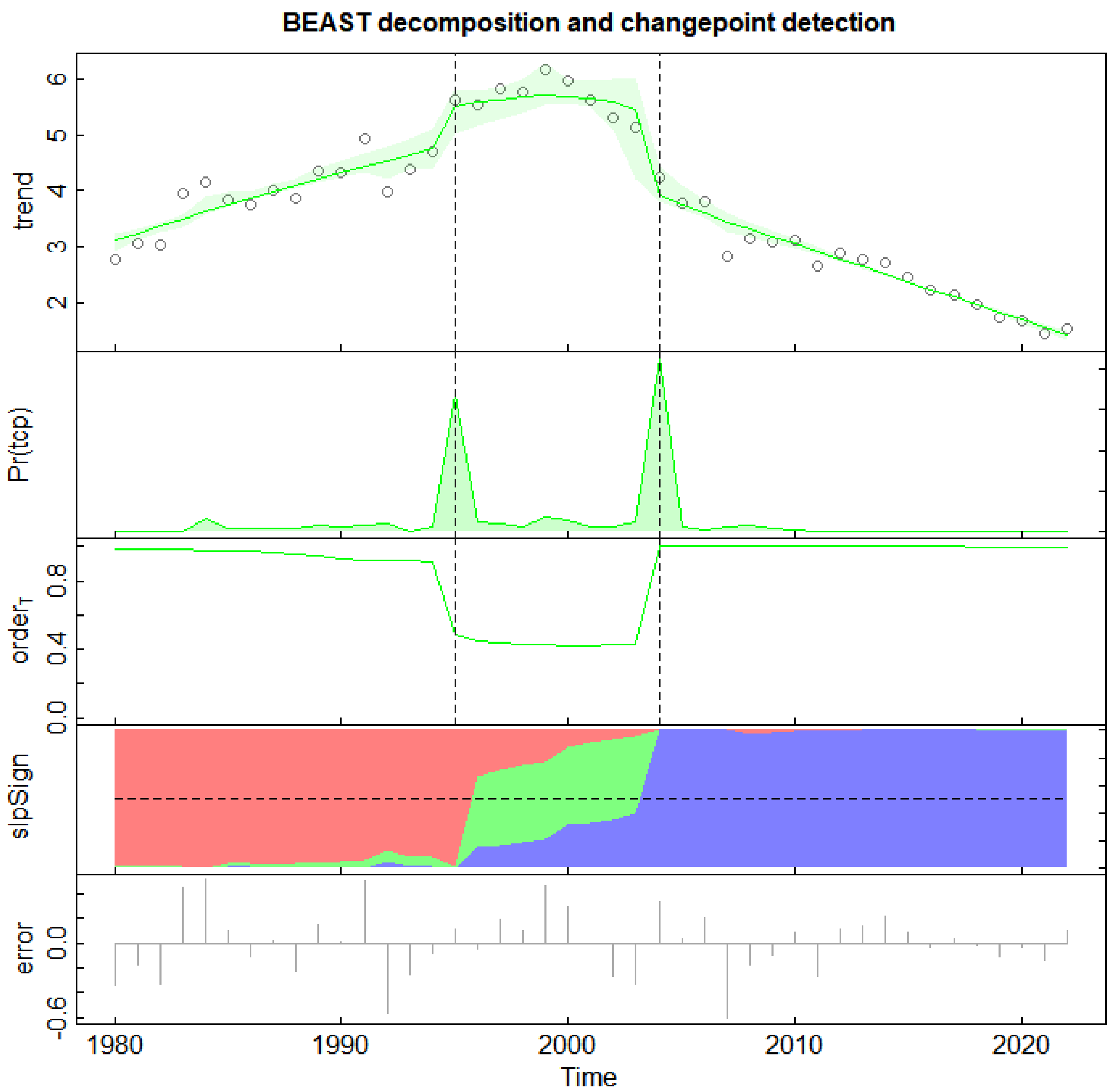


**Figure S1.** Results of BEAST of female homicides time series in São Paulo from 2000 to 2022.

The `print(out)` function summarizes and prints the results obtained from the BEAST time series decomposition and segmentation, as shown below.

```
> print(out)

#####################################################################
#                       Seasonal  Changepoints                        #
#####################################################################
No seasonal/periodic component present (i.e., season='none')

#####################################################################
#                       Trend  Changepoints                           #
#####################################################################
.-------------------------------------------------------------------.
| Ascii plot of probability distribution for number of chgpts (ncp) |
.-------------------------------------------------------------------.
|Pr(ncp = 0 )=0.000|*                                               |
|Pr(ncp = 1 )=0.080|******                                          |
|Pr(ncp = 2 )=0.691|*********************************************** |
|Pr(ncp = 3 )=0.196|**************                                  |
|Pr(ncp = 4 )=0.031|***                                             |
|Pr(ncp = 5 )=0.003|*                                               |
|Pr(ncp = 6 )=0.000|*                                               |
|Pr(ncp = 7 )=0.000|*                                               |
|Pr(ncp = 8 )=0.000|*                                               |
|Pr(ncp = 9 )=0.000|*                                               |
.-------------------------------------------------------------------.
|    Summary for number of Trend ChangePoints (tcp)                 |
.-------------------------------------------------------------------.
|ncp_max    = 9    | MaxTrendKnotNum: A parameter you set           |
|ncp_mode   = 2    | Pr(ncp= 2)=0.69: There is a 69.1% probability  |
|                  | that the trend component has  2 changepoint(s).|
|ncp_mean   = 2.19 | Sum{ncp*Pr(ncp)} for ncp = 0,...,9             |
|ncp_pct10  = 2.00 | 10% percentile for number of changepoints      |
|ncp_median = 2.00 | 50% percentile: Median number of changepoints  |
|ncp_pct90  = 3.00 | 90% percentile for number of changepoints      |
.-------------------------------------------------------------------.
| List of probable trend changepoints ranked by probability of      |
| occurrence: Please combine the ncp reported above to determine    |
| which changepoints below are practically meaningful               |
'-------------------------------------------------------------------'
|tcp#              |time (cp)                  |prob(cpPr)          |
|------------------|---------------------------|--------------------|
|1                 |2004.000000                |0.94046             |
|2                 |1995.000000                |0.77420             |
|3                 |1992.000000                |0.11254             |
|4                 |1984.000000                |0.09895             |
.-------------------------------------------------------------------.
```

This output shows that there is a 69.1% probability that the trend component has two change points (the mode for the probability distribution for number of change points, denoted by `ncp_mode`). The locations where these two change points occur and the probabilities associated with each change point are 2004 (94.06%) and 1995 (77.42%).

**Statistical details of APC estimation**

When the BEAST model suggests an approximately linear trend in an interval on the time series, it is possible to calculate the correspondent annual percentage rate change (APC)[7]. APC is defined as

$$APC = \frac{\gamma_{j+1} - \gamma_j}{\gamma_j},$$

where $\gamma_j$ denotes the expected rate at time $j$. If we assume that $\log(\gamma_j)$ is linear over the entire time interval $[t_0, t_1]$, then

$$\log \gamma_j = a + bt_j,$$

where $a$ and $b$ are constants. Note that

$$\frac{\gamma_{j+1}}{\gamma_j} = \frac{e^{a+bt_{j+1}}}{e^{a+bt_j}} = e^{b(t_{j+1}-t_j)} = e^b$$

when $t_{j+1} - t_j = 1$, i.e. when considering a change of one time unit. Therefore,

$$APC = \frac{\gamma_{j+1} - \gamma_j}{\gamma_j} = \frac{\gamma_{j+1}}{\gamma_j} - 1 = e^b - 1,$$

where $e$ is the base of the natural logarithm and $b$ is the angular coefficient of the Prais-Winsten regression model.

We let $y_t$ denote observations of a variable at $n$ time points $t = 1,\ldots,n$. A linear regression model is given by

$$\log y_t = a + bt + \epsilon_t,$$

where $a$ and $b$ are unknown coefficients and $\epsilon_t$ is a mean zero error representing other determinants of the outcome. We assume that $t$ is independent of $\epsilon_t$. The Prais-Winsten method involves estimating the correlation between the error at $t$ and $t - 1$, assuming that

$$\epsilon_t = \phi_1 \epsilon_{t-1} + \eta_t,$$

where $\phi_1$ is an autoregressive (AR) parameter with $|\phi_1| < 1$ and $\eta_t$ are independent disturbances with zero mean and variance $\sigma_\eta^2$. The constraint $\phi_1$ ensures that the process is stationary, that is, the covariance between $\epsilon_t$ and $\epsilon_{t+h}$ is independent of $t$, and therefore

that the variance remains constant over time. In our article, this analysis used the function `prais_winsten` of the `prais` package in R.

For example, to estimate the APC of the of female homicide rates time series between $t_0$ = 1980 and $t_1$ =1994, we can use the following R code:

```
# APC estimation based on the Prais-Winsten regression model
# 1980 to 1994
library(prais)
pw <- c()
ay <- 1980
by <- 1994
NY    <- length(ay:by)
seqt  <- (ay-1979):(by-1979)
pw_sample <- data.frame("x" = wt$ANO[seqt], "y" = wt$SP[seqt],
               "time" = seqt)
pw    <- prais_winsten(log(y) ~ x, data = pw_sample, index = "time")
a     <- summary(pw)
b1    <- a$coefficients[2,1]    # angular coefficient
se    <- a$coefficients[2,2]    # standard errors
APC   <- exp(b1)-1              # APC
L_APC <- exp(b1-se*qt(1-0.05/2,NY-1))-1
U_APC <- exp(b1+se*qt(1-0.05/2,NY-1))-1
message("APC ",round(100*APC,2)," 95%CI (",round(100*L_APC,2),
        " , ",round(100*U_APC,2),")")
```

In this R code, the variable `wt$ANO` is a vector from the data frame `wt` containing the years 1980 to 2022.

The following output is then obtained for the Prais-Winsten regression model analysis:

```
Call:
prais winsten(formula = log(y) ~ x, data = pw sample, index = "time")

Residuals:
      Min        1Q    Median        3Q       Max 
-0.133876 -0.057356 -0.002484  0.053558  0.158118 

AR(1) coefficient rho after 6 iterations: 0.2012

Coefficients:
              Estimate Std. Error t value Pr(>|t|)    
(Intercept) -60.152761  12.923666   -4.654 0.000451 ***
x             0.030957   0.006504    4.760 0.000373 ***
---
Signif. codes:  0 '***' 0.001 '**' 0.01 '*' 0.05 '.' 0.1 ' ' 1

Residual standard error: 0.09134 on 13 degrees of freedom
```

```
Multiple R-squared:  0.428,     Adjusted R-squared:  0.384
F-statistic: 9.727 on 1 and 13 DF,  p-value: 0.008146

Durbin-Watson statistic (original): 1.473
Durbin-Watson statistic (transformed): 1.856
```

We can note that the estimated values for the parameters $\phi_1$ and $b$ are 0.2012 and 0.030957, respectively. Thus, the APC is estimated to be

$$APC = (e^{0.030957} - 1)100\% \cong 3.14\%.$$

The lower and upper limits of the 95% confidence interval for the APC can be obtained using the following expressions, respectively:

$$[\exp(b - se(b) \times t_{(1-0.05/2,df)}) - 1]100\%$$

and

$$[\exp(b + se(b) \times t_{(1-0.05/2,df)}) - 1]100\%$$

In these expressions, se($b$) is an estimate of the standard error of $b$. In the R output, we have se($b$) equal to 0.006504. In addition, $t_{(1-0.05/2,df)}$ is the (1–0.05/2) percentile of a Student's t distribution with degrees of freedom (df) given by $n - 1$, where $n$ is the length of the interval. This percentile is obtained using the R function `qt`.

The R code above also provides the following:

```
APC 3.14 95%CI (1.72 , 4.59)
```

Thus, assuming that the annual rate changes are constant throughout the time interval [1980, 1994], we estimate an annual percentage rate change of 3.14% for this period.

R program version 4.1.1 was used for all these analyses.

## References


1. Li J, Li ZL, Wu H, You N. Trend, seasonality, and abrupt change detection method for land surface temperature time-series analysis: Evaluation and improvement. Remote Sens Environ 2022; 280, 113222. https://doi.org/10.1016/j.rse.2022.113222

2. Dan'azumi S, Mamudu L, Aldrees A. Climate change detection and attribution: Bayesian estimation of abrupt change, seasonality and trend model, and Mann–Kendall trend test approaches. J Water Clim Chang 2025;16(5):1895-911. https://doi.org/10.2166/wcc.2025.004
3. Yang L, Wang C, Zhou P, Xie N, Tian M, Wang K. Change point detection in brucellosis time series from 2010 to 2023 in Xinjiang China using the BEAST algorithm. Scientific Reports 2025;15(1):3830. https://doi.org/10.1038/s41598-025-88508-0
4. Kim HJ, Chen HS, Byrne J, Wheeler B, Feuer EJ. Twenty years since Joinpoint 1.0: Two major enhancements, their justification, and impact. Stat Med 2022;41(16):3102-30. https://doi.org/10.1002/sim.9407
5. Zhao K, Wulder MA, Hu T, Bright R, Wu Q, Qin H, Li Y, Toman E, Mallick B, Zhang X, Brown M. Detecting change-point, trend, and seasonality in satellite time series data to track abrupt changes and nonlinear dynamics: A Bayesian ensemble algorithm. Remote Sens. Environ 2019; 232: 111181. https://doi.org/10.1016/j.rse.2019.04.034
6. Yang L, Wang C, Zhou P, Xie N, Tian M, Wang K. Change point detection in brucellosis time series from 2010 to 2023 in Xinjiang China using the BEAST algorithm. Scientific Reports 2025;15(1):3830. https://doi.org/10.1038/s41598-025-88508-0
7. Clegg LX, Hankey BF, Tiwari R, Feuer EJ, Edwards BK. Estimating average annual per cent change in trend analysis. Stat Med 2009;28(29):3670-82. https://doi.org/10.1002/sim.3733
